# Band-Gap Engineering of High-Entropy Fluorite Metal Oxide Nanoparticles Facilitated by Pr^3+^ Incorporation by Gel Combustion Synthesis

**DOI:** 10.3390/gels11020117

**Published:** 2025-02-06

**Authors:** Mariappan Anandkumar, Kannan Pidugu Kesavan, Shanmugavel Sudarsan, Olga Vladimirovna Zaitseva, Ahmad Ostovari Moghaddam, Daria Valerevna Iarushina, Evgeny Alekseevich Trofimov

**Affiliations:** 1 High-Entropy Materials Research Laboratory, South Ural State University, 454080 Chelyabinsk, Russia; 2 Department of Physics, PSG Institute of Technology and Applied Research, Coimbatore 641 062, India; 3 Laboratory of Problems of Recycling Modern Multicomponent Materials with Complex Structure, South Ural State University, 454080 Chelyabinsk, Russia; 4 Department of Industrial and Civil Engineering, South Ural State University, 454080 Chelyabinsk, Russia; 5 Department of Applied Mathematics, National Research University Higher School of Economics, 101000 Moscow, Russia; 6 Department of Materials Science, Physical and Chemical Properties of Materials, South Ural State University, 454080 Chelyabinsk, Russia

**Keywords:** high-entropy oxide, gel combustion, band-gap, solid solution, absorbance, fluorite oxide

## Abstract

Tailoring the bandgap of a material is necessary for improving its optical properties. Here, the optical bandgap of high-entropy oxide Ce_0.2_Gd_0.2_Sm_0.2_Y_0.2_Zr_0.2_O_2-δ_ (HEO) nanoparticles was modified using Pr^3+^. Various concentrations of Pr^3+^ (x = 0, 0.01, 0.02, 0.05, 0.075, 0.1, 0.15) were incorporated into the host high-entropy oxide using a gel combustion synthesis. After the gel combustion step, the powders were heat-treated at various temperatures (650 °C, 800 °C, 950 °C) for 2 h. The obtained Pr^3+^-incorporated HEO powders were characterized using X-ray diffraction (XRD), field emission scanning electron microscopy (FESEM), and UV–visible spectroscopy. The results indicate that, when the samples are calcined at 950 °C, a single-phase cubic fluorite structure is obtained without any phase separation or impurity. The optical absorbance red-shifts to higher wavelengths when the concentration of Pr^3+^ is increased. This reduces the bandgap of the material from 3.15 eV to 1.87 eV for Pr^3+^ concentrations of x = 0 (HEO-0) and x = 0.15 (HEO-6), respectively. The obtained HEOs can be suitable candidates for photocatalytic applications due to their absorbance in the visible region.

## 1. Introduction

High-entropy materials have made a significant progress in materials science research, demonstrating excellent properties and a wide range of functional applications [1,2,3]. High-entropy materials, including alloy systems first explored in previous studies, and other material classes such as ceramics were investigated [1,4]. The entropy effect is one of the four core effects pertaining to typical high-entropy materials, playing a significant role in stabilizing a single-phase solid solution. Therefore, a variety of chemical compositions can be prepared using the concept of high entropy. However, higher entropy does not necessarily lead to the formation of a single phase, and other factors are involved, which is a matter of debate.

In recent years, the focus on high entropy oxides has increased due to their tailorable properties, including magnetic [5,6], electrical [7], thermal [8], photoluminescent [9], electromagnetic [10], and better functional applications [11,12,13,14,15]. In the case of photocatalytic applications, the bandgap of a material determines the performance of a photocatalyst [16,17,18,19]. Natural sunlight contains ~5% UV, ~43% visible and ~52% infrared light, and most of the metal oxides have optical absorbance in the UV region [20,21]. Therefore, the bandgap of a material decides which wavelength of the available solar spectrum is effectively absorbed to generate electron–hole pairs. Usually, a material with lower bandgap is preferred, so that the photocatalytic reaction can be performed using natural sunlight or a visible light source instead of using a harmful UV–light source. In order to achieve this, various strategies are utilized, such as varying the particle size [16,22], morphology [23,24], adding dopants [25,26], forming heterojunctions [27,28], optimizing synthesis conditions [29,30,31], etc. We have selected bandgap engineering by doping as it can be easily controlled by optimizing the concentration of the dopant. For instance, the bandgap of typical CeO_2_ is 3.4 eV, allowing it to form electron–hole pairs solely using ultraviolet energy. This limits the use of UV-absorbing photocatalysts such as TiO_2_, CeO_2_, ZrO_2_, HfO_2_, etc., in the visible spectrum. To tailor the absorbance of a photocatalyst in visible regions, a variety of dopants such as Pr [32], Fe [33], Cu [34], Ni [35], Bi [36], N [37] can be doped into the host oxide. This decreases the bandgap of the material improving the photocatalyst performance. In addition, other modification strategies such as forming heterojunctions using ZnMn_2_O_4_, BaTiO_3_, ZnO, CdS, Ag_2_S [38,39], and carbon-based materials [40,41] were employed to optimize the electron–hole recombination behavior facilitating superior photocatalytic activity.

In general, the synthesis technique plays a crucial role in tuning the material properties, which impact its functional properties and applications [42,43,44,45]. Various synthesis techniques such as chemical co-precipitation, solid-state, emulsion, gel combustion, hydrothermal, microwave-assisted were available to synthesize a variety of nanomaterial systems ranging from metals to oxides. Among them, gel combustion is one of the most widely used synthesis for obtaining a variety of nanoparticles. Gel combustion synthesis combines the advantages of the sol–gel technique and combustion technique. In a sol–gel technique, controlled particle size and stoichiometry are obtained. Similarly, combustion synthesis forms nanoparticles with defects in terms of oxygen vacancies (for metal oxide systems).

Therefore, in gel combustion synthesis, one can tailor the particle size and defects, which play a critical role in functional applications [46]. In addition, the use of glycine can act as a chelating agent, as well as fuel, during the combustion step, which is advantages [46]. Kumar et al. prepared GdAlO_3_:Dy^3+^ nanophosphors using a gel combustion with urea as a fuel and the resultant oxide was used for white light applications [47]. Chavarriaga et al. followed a gel-combustion synthesis to synthesize CoFe_2_O_4_, ZnFe_2_O_4_, and MgFe_2_O_4_ oxides using 6-aminohexanoic acid as a fuel and explored its magnetic properties [48]. Similarly, Portakal-Uçar et al. used a gel combustion technique using citric acid to prepare Zn_2_SiO_4_ co-activated by Ce^3+^ and Mn^2+^ ions and investigated its luminescence properties [49]. Kumar et al. synthesized Ni_0.6_Zn_0.4_Gd_y_Fe_2-y_O_4_ (y = 0, 0.1, 0.15, and 0.2) nanoferrite by gel combustion using citate and explored its electrical conductivity [50].

Therefore, we are more interested in investigating the use of gel combustion synthesis route to form high-entropy oxide nanoparticles. In the present study, we use a gel combustion synthesis to produce various compositions of Pr^3+^-incorporated high-entropy Ce_0.2_Gd_0.2_Sm_0.2_Y_0.2_Zr_0.2_O_2-δ_ oxide nanoparticles. The rationale for selecting the current composition as a host material lies in the wide bandgap nature of Ce_0.2_Gd_0.2_Sm_0.2_Y_0.2_Zr_0.2_O_2-δ_ oxide system [29]. Therefore, these materials exhibit photocatalytic activity in in the UV-region, which limits their use as flexible photocatalysts in the visible region. The use of the visible-light-activated photocatalyst will help to harvest naturally available sunlight and visible light source, enhancing versatility of the photocatalyst. Therefore, with the help of Pr^3+^ incorporation, we are more interested in narrowing the bandgap of Ce_0.2_Gd_0.2_Sm_0.2_Y_0.2_Zr_0.2_O_2-δ_ oxide to absorb radiation in the visible region. In the same way, Pr^3+^ was selected in the current study due to its mixed valance state, including 3+ and 4+. Due to this, when Pr^3+^ was incorporated into a metal oxide lattice, defects in terms of oxygen vacancies were created to balance the charge imbalance in the system [51]. Likewise, the phase evolution of the prepared nanoparticles and the influence of Pr^3+^ incorporation on the optical properties were investigated.

## 2. Results and Discussion

### 2.1. Structural Investigation

**Scheme 1:**
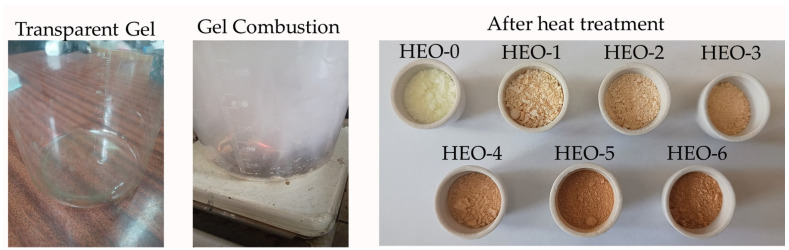
Steps involved in the synthesis of HEO and Pr^3+^-incorporated HEO nanoparticles prepared by gel combustion synthesis. (**Left**) Transparent gel formed after evaporation of water; (**middle**) initiation of gel combustion; and (**right**) visual appearance of HEO and Pr^3+^-incorporated HEO nanoparticles after heat treatment. HEO-0 (pure, x = 0), HEO-1 (x = 0.01), HEO-2 (x = 0.02), HEO-3 (x = 0.05), HEO-4 (x = 0.075), HEO-5 (x = 0.1), and HEO-6 (x = 0.15).

Scheme 1 describes the synthesis process involving gel combustion using glycine as a chelating agent and fuel. When the metal salts and glycine are mixed together with de-ionized water, the glycine molecules interact with the metal ions in the solution, forming metal complexes. The formed metal complexes prevent the selective precipitation of metal ions in the solution, ensuring higher homogeneity of the resultant oxides. During heating, the water evaporates consistently, resulting in a transparent gel. As mentioned earlier, glycine acts as a fuel during the combustion of gel, exhibiting a dual role. During the combustion step, the resultant transparent gel swells, transforming into a foam, followed by auto combustion. This process releases a large volume of gas, such as CO_2_, N_2_, H_2_O, and O_2_, resulting in the formation of porous high-entropy oxide nanoparticles [52]. Heat treatment was conducted in order to remove the any unreacted glycine and carbon produced during the synthesis step.

**Figure 1:**
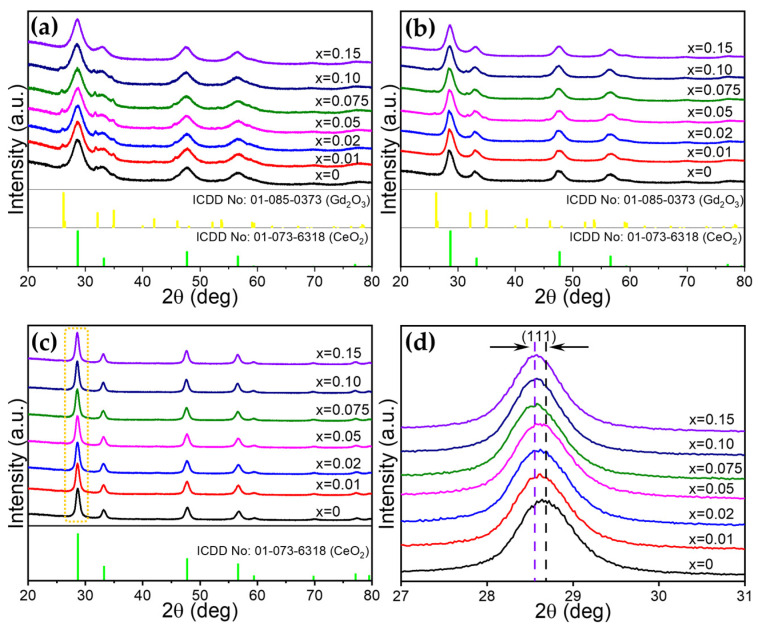
XRD patterns of HEO and Pr^3+^-incorporated HEO nanoparticles prepared by gel combustion synthesis calcined at various temperatures: (**a**) 650 °C, (**b**) 800 °C, and (**c**) 950 °C. (**d**) Enlarged XRD pattern of the (111) plane for various samples heat-treated at 950 °C, showing a peak shift when different concentrations of Pr^3+^ were incorporated into the HEO lattice. HEO-0 (pure, x = 0), HEO-1 (x = 0.01), HEO-2 (x = 0.02), HEO-3 (x = 0.05), HEO-4 (x = 0.075), HEO-5 (x = 0.1), and HEO-6 (x = 0.15).

The phase evolution of HEO and Pr^3+^-incorporated HEO nanoparticles was investigated at various calcination temperatures, such as 650 °C, 800 °C, and 950 °C, using XRD. The plot is displayed in Figure 1. When the samples are calcined at 650 °C and 800 °C (Figure 1a,b), all the broad peaks centered around 28°, 33°, 47°, 56° are indexed to a cubic fluorite structure (ICDD No: 01-085-0373, CeO_2_). In addition, minor impurity phases are observed, pertaining to Gd_2_O_3_ (ICDD No: 01-073-6318). This confirms that a single-phase fluorite oxide is not favorable and needs high temperature calcination. When the calcination temperature was increased to 900 °C, the impurity phase disappeared, and a single-phase fluorite oxide was formed. In addition, when the concentration of Pr^3+^ was increased (x = 0, 0.01, 0.02, 0.05, 0.075, 0.1, 0.15), we observed that the main peak shifted to lower angles, indicating successful Pr^3+^ incorporation into the HEO lattice without forming an additional phase (Figure 1d).

**Figure 2:**
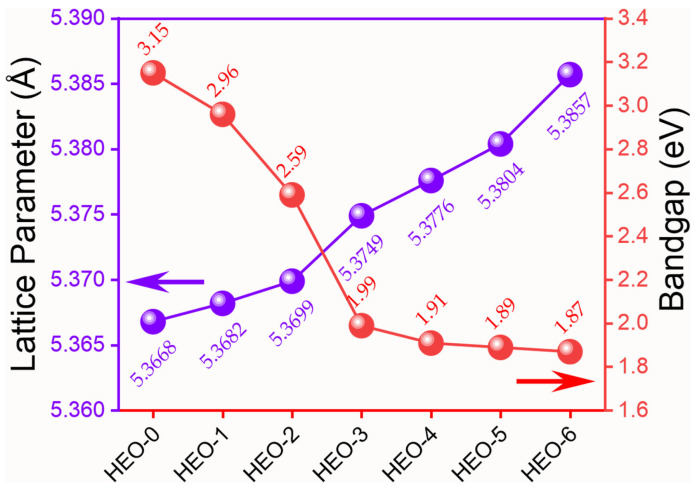
Estimated bandgap and lattice parameter of HEO and Pr^3+^-incorporated HEO nanoparticles prepared by gel combustion synthesis calcined at 950 °C. HEO-0 (pure, x = 0), HEO-1 (x = 0.01), HEO-2 (x = 0.02), HEO-3 (x = 0.05), HEO-4 (x = 0.075), HEO-5 (x = 0.1), and HEO-6 (x = 0.15).

Rietveld refinement (FullProf Suit, Version: January 2021) was performed in order to fit the experimental XRD pattern using a simulated pattern featuring cubic fluorite as a crystal structure (space group: Fm-3m, cubic) (Figure S1) [53]. The atomic positions of metal cations were (0,0,0), while the oxygen anion was (0.25,0.25,0.25). During the structural refinement process, parameters such as background, scale factor, half-width parameters, lattice parameter, atomic fractional position coordinates, and thermal parameters are optimized. From the Rietveld refinement results, it is evident that the experimental data aligned well with the simulated pattern, indicating the formation of a single-phase cubic fluorite structure (Figure S1). The estimated lattice parameter from the Rietveld refinement results is tabulated in Table 1. For the HEO-0 sample, the lattice parameter is 5.3368 Å. With an increase in Pr^3+^ concentration, the lattice parameter increases from 5.3682 Å (HEO-1) to 5.3857 Å (HEO-6). The incorporation of larger Pr^3+^ ionic radii (1.126 Å) into the HEO lattice makes the lattice expand, which can be supported by an increase in the lattice parameter (Figure 2). In contrast, the micro-strain starts to reduce drastically from 0.00392 (HEO-1) to 0.00078 (HEO-6) with an increase in Pr^3+^ incorporation, which is unusual. This is expected to be due to the relaxation of the lattice rather than straining. For example, for the HEO-6 sample, based on the elemental composition, all the principal elements involved are nearly equiatomic, and the influence of Pr^3+^ does not significantly affect the HEO lattice, as expected at lower concentrations. A similar effect can be observed in the optical properties, which will be discussed in a later section.

**Figure 3:**
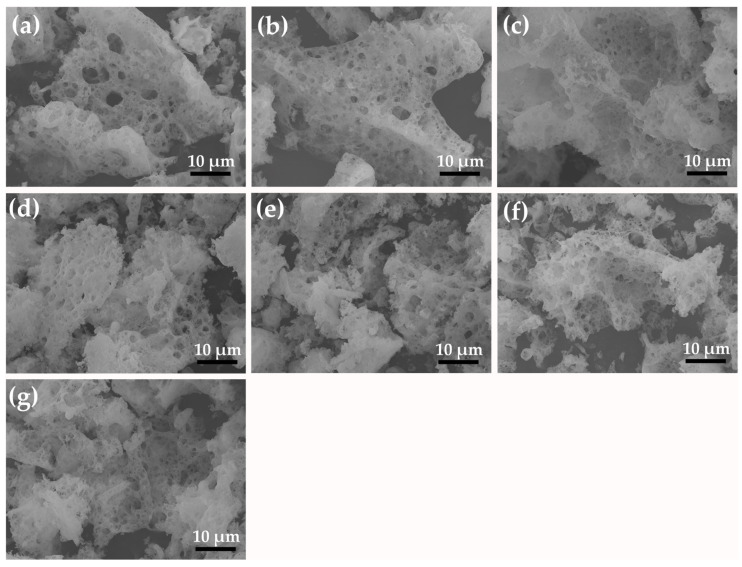
FESEM image of HEO and Pr^3+^-incorporated HEO nanoparticles prepared by gel combustion synthesis and calcined at 950 °C: (**a**) HEO-0, (**b**) HEO-1, (**c**) HEO-2, (**d**) HEO-3, (**e**) HEO-4, (**f**) HEO-5, and (**g**) HEO-6.

The morphological nature of HEO and Pr^3+^-incorporated HEO nanoparticles prepared by gel combustion synthesis was studied using an FESEM and is displayed in Figure 3. As we know, the gel combustion synthesis resulted in a spongy-like textured nanoparticles possessing micro- and nano-sized pores. During the gel combustion step, self-combustion occurs, releasing a large volume of gases and resulting in porous nanostructures. With an increase in Pr^3+^ concentration, there are no significant changes in the morphology and the nature of the pores. However, the crystallite size estimation from the XRD pattern (Table 1) is affected, which can enhance the catalytic and photocatalytic properties.

**Figure 4:**
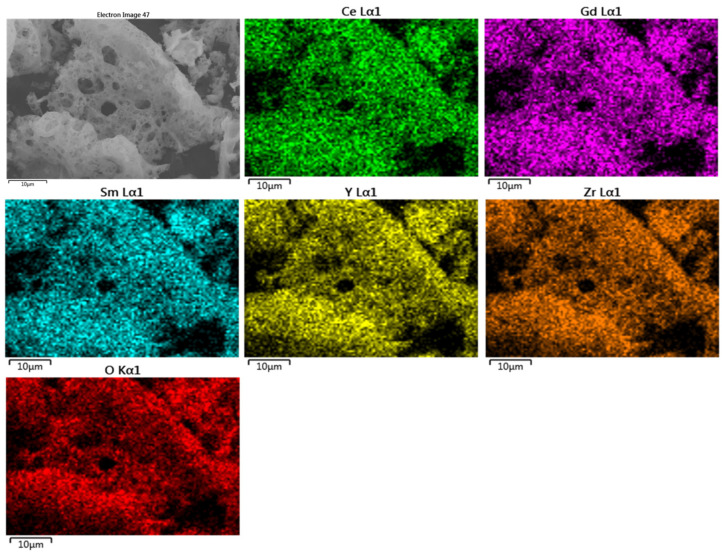
Elemental mapping of HEO-0 nanoparticles.

**Figure 5:**
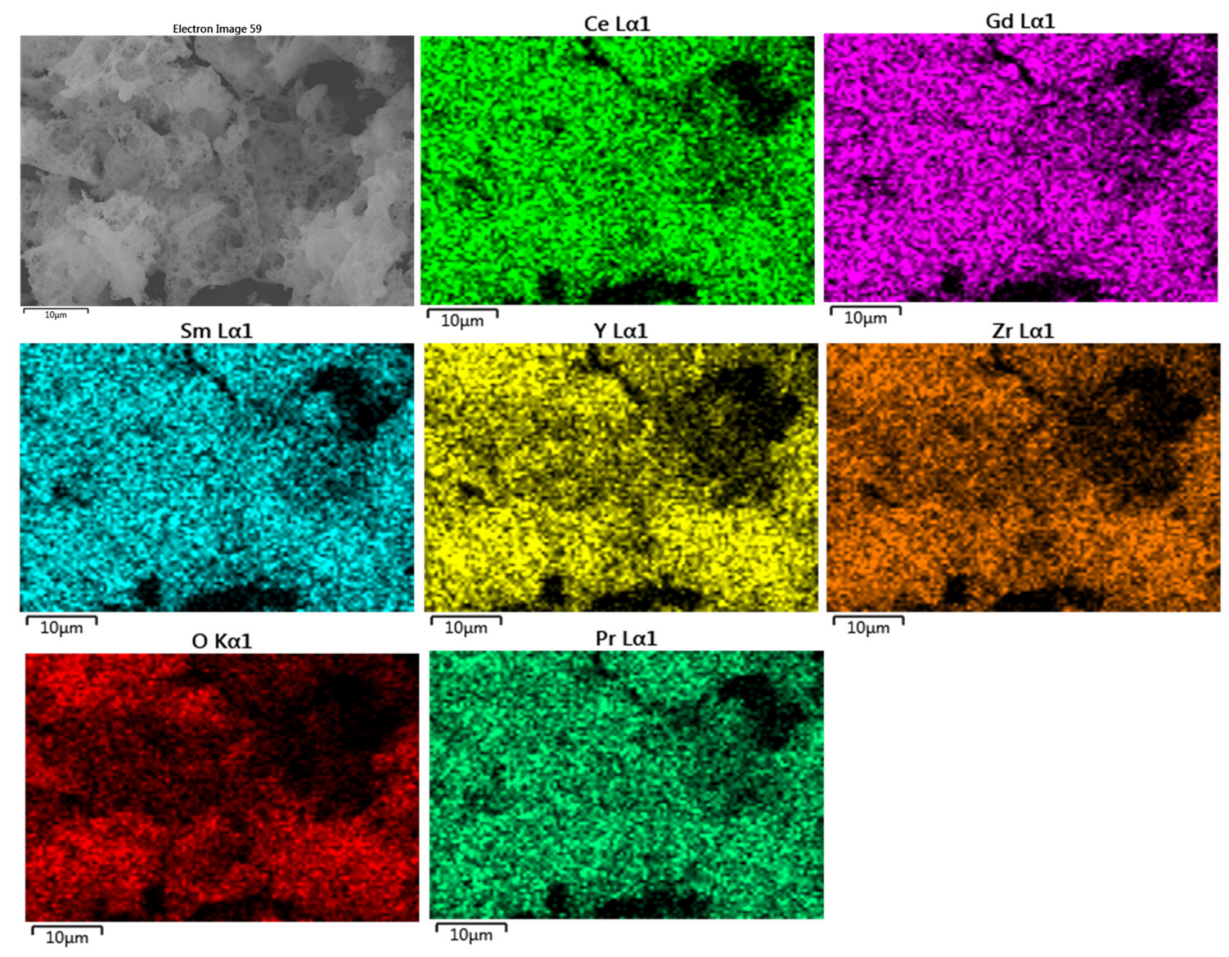
Elemental mapping of HEO-6 nanoparticles.

The elemental mapping of HEO and Pr^3+^-incorporated HEO nanoparticles prepared by gel combustion synthesis is carried out in order to probe the elemental distribution of various metal cations within the particles (Figure 4 and Figure 5). The results show that all the individual metal cations are evenly distributed within the particles, which is one of the advantages of using a gel combustion synthesis. The mean elemental quantification was estimated from the EDS point scan averaging from 15 different locations and is tabulated in Table 2. The results conclude that the concentration of individual metal cations is similar across all locations and uniformly distributed, as supported by elemental mapping, indicating better sample preparation.

### 2.2. Bandgap Engineering of HEO by Pr^3+^ Incorporation

**Figure 6:**
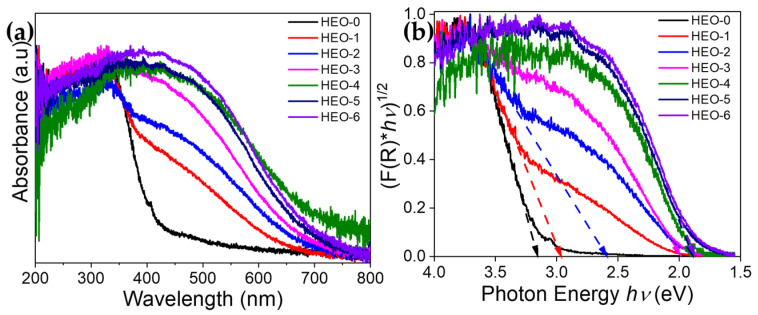
UV–visible absorption spectra of HEO and Pr^3+^-incorporated HEO (**a**) and its corresponding Kubelka–Munk plot to estimate the bandgap of the prepared oxides (**b**): HEO-0 (pure, x = 0), HEO-1 (x = 0.01), HEO-2 (x = 0.02), HEO-3 (x = 0.05), HEO-4 (x = 0.075), HEO-5 (x = 0.1), and HEO-6 (x = 0.15).

The UV–visible absorption spectra for all the Pr^3+^-incorporated HEO systems is shown in Figure 6. It is observed that for all the Pr^3+^-incorporated HEO samples, the absorbance values start to increase from 700 nm. However, in case of pure HEO, the absorbance value blue-shifted to lower wavelengths. This is visually observed by the color of the nanoparticles (Scheme 1). Pure HEO has a pale-yellow color, which gradually transformed to dark brown with an increase in Pr^3+^ concentration. As a result of Pr^3+^ incorporation, better absorbance in the visible region is achieved. The absorbance of all the samples can be correlated to the charge transfer transitions existing between O^2−^ and Me^3+/4+^ [29].

The bandgap values estimated from the K-M plot (Figure 6b) are tabulated in Table 1. For the pure HEO sample, the bandgap value of 3.15 eV is obtained. However, bandgap narrowing occurred when Pr^3+^ was incorporated, and the bandgap values reduced to 1.87 eV (HEO-6). One can observe that at a lower Pr^3+^ concentration, there is a larger variation in bandgap values for the samples between HEO-0 and HEO-2, and the change is marginal for the samples between HEO-3 and HEO-6 (Figure 2). Overall, the change in bandgap values due to Pr^3+^ incorporation can be explained as follows. One possible reason is the role of oxygen vacancies or defects present in the system [54]. For example, the HEO sample contains both trivalent and tetravalent cations. When tetravalent cations are replaced by trivalent cations, due to charge imbalance, point defects in terms of oxygen vacancies are created in the crystal lattice, as denoted by the Kröger–Vink notation [55] (Equation (1))

(1) $$O_{o}^{x}+2{Me}_{Me}^{x}⟶\;V_{o}^{••}+2{Me}_{Me}^{\prime }+\frac{1}{2}O_{2}$$

where $O_{o}^{x}$ and ${Me}_{Me}^{x}$ are tetravalent cations present in their respective lattice position, while $V_{o}^{••}$ is the oxygen vacancy at the oxygen lattice position, and ${Me}_{Me}^{\prime }$ is the trivalent cation occupying the tetravalent position. Therefore, metal cations such as Ce^4+^ and Pr^4+^ can be reduced to Ce^3+^ and Pr^3+^, respectively since they possess multiple oxidation states, such as 3+ and 4+. The presence of oxygen vacancies in Pr^3+^-incorporated HEOs shifts the bandgap to the visible region of the electromagnetic spectrum. However, due to limited characterization capability, the exact quantification of oxygen vacancies will be carried out in our future investigation.

**Figure 7:**
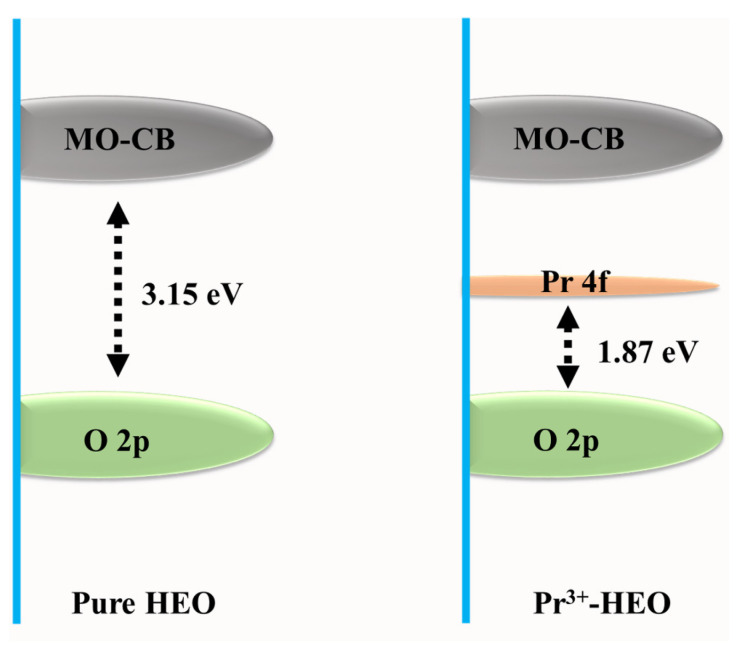
Proposed band diagram of pure HEO and Pr^3+^-incorporated HEO. Pr^3+^ incorporation alters the bandgap of the HEO nanoparticle by creating an intermediate energy level, narrowing the bandgap of the material (MO-CB: metal oxide conduction band).

Reports clarify that apart from oxygen vacancies, there are other possibilities for the narrowing of the bandgap, such as the creation of intermediate energy levels due to Pr^3+^ incorporation [56]. Therefore, in our case, the bandgap of HEO was modified by introducing an intermediate energy level by Pr^3+^ incorporation (Figure 7) [57,58]. This narrows the overall bandgap of the HEO system. Therefore, the bandgap of high-entropy oxides can be tailored by Pr^3+^ incorporation, which is beneficial for applications such as photocatalysis where narrow bandgap values can absorb visible light. This can enhance the photocatalytic activity just by utilizing natural sunlight or visible light without the need of using extremely harmful UV-light sources. The current study will pave the way to designing new photocatalytic systems and tailoring their optical properties.

## 3. Conclusions

This study aimed to synthesize new high-entropy oxide Ce_0.2_Gd_0.2_Sm_0.2_Y_0.2_Zr_0.2_O_2-δ_ (HEO) nanoparticles by incorporating different concentrations of Pr^3+^ (x = 0, 0.01, 0.02, 0.05, 0.075, 0.1, 0.15). A simple gel combustion synthesis, using glycine as a chelating agent and fuel, was used. A single-phase fluorite structure was obtained when the as-synthesized samples were calcined at 950 °C without any phase separation. In all cases, Pr^3+^ was successfully incorporated into the HEO lattice. Pr^3+^ incorporation had an effect on the particle size, lattice parameters, and bandgap of the material. When Pr^3+^ was introduced into the HEO lattice, an intermediate energy level was created, narrowing the bandgap of HEO. The bandgap of HEO-0 was 3.15 eV, while after Pr^3+^ incorporation, the bandgap was narrowed to 1.87 eV for HEO-6. Overall, the prepared nanoparticles can be suitable candidates for photocatalytic applications due to their absorbance in the visible region. The current study will give researchers an opportunity to explore the functional applications of HEO nanoparticles.

## 4. Materials and Methods

### 4.1. Raw Materials

Yttrium (III) nitrate hexahydrate (Y(NO_3_)_3_•6H_2_O, 99.8 %, Sigma-Aldrich, Moscow, Russia), Gadolinium (III) nitrate hexahydrate (Gd (NO_3_)_3_•6H_2_O, 99.9%, Sigma-Aldrich, Moscow, Russia), Praseodymium (III) nitrate hexahydrate (Pr(NO_3_)_3_•6H_2_O, 99.9%, Sigma-Aldrich, Moscow, Russia), Samarium (III) nitrate hexahydrate (Sm(NO_3_)_3_•6H_2_O, 99.9%, Sigma-Aldrich, Moscow, Russia), Cerium (III) nitrate hexahydrate (Ce (NO_3_)_3_•6H_2_O, 99.9%, Sigma-Aldrich, Moscow, Russia), Zirconium (IV) oxynitrate dihydrate (ZrO(NO_3_)_2_•2H_2_O, 99%, Sigma-Aldrich, Russia), and glycine (NH_2_CH_2_COOH, ≥99.0% (NT), BioUltra, Sigma-Aldrich, Moscow, Russia) were used. All the materials were used as received, without any further purification. Deionized (DI) water was used throughout the study.

### 4.2. Synthesis

A simple gel combustion method was utilized for the synthesis of pure and Pr^3+^-incorporated high-entropy oxide nanoparticles. Initially, the required amounts of individual salts were weighed and added to the beaker. The concentration of total metal cations was fixed at 0.005 moles throughout the experiments. Minimal DI water was added to dissolve the salts until the formation of a clear solution. Next, glycine (the molar ratio of metal cation to glycine was 1:1.4) was added and dissolved until a clear solution was obtained. A transparent gel was formed when the resultant solution was heated on a hotplate (130 °C) to evaporate the water. Next, the temperature of the hot plate was raised to 320 °C to initiate the gel combustion reaction. After the combustion reaction was complete, the resultant powder was calcined in a muffle furnace at various temperatures, such as 650 °C, 800 °C, and 950 °C for 2 h to decompose any unreacted glycine and precursor. In the case of Pr^3+^ incorporation, various amounts of Pr^3+^ precursor were added to the above step to form various Pr^3+^-incorporated HEOs. The sample code of pure and Pr^3+^-incorporated HEOs is HEO-0 (pure, x = 0), HEO-1 (x = 0.01), HEO-2 (x = 0.02), HEO-3 (x = 0.05), HEO-4 (x = 0.075), HEO-5 (x = 0.1), and HEO-6 (x = 0.15).

### 4.3. Characterization

The phase evolution of pure and Pr^3+^-incorporated HEOs was investigated using XRD (Rigaku Ultima IV, Rigaku Corporation, Akishima, Japan), consisting of a Cu target (K_α_ radiation with a λ = 1.54 Å). The sample was scanned from 20 to 80° (scan speed of 1° per minute) whose resolution is 0.0001°. FESEM images were captured using a JEOL (JEOL JSM-7001F, JEOL, Peabody, MA, USA) microscope operated at 20 kV, and the elemental composition was estimated using an energy-dispersive X-ray spectroscopy (EDS) detector (Oxford INCA X-max 80, Oxford Instruments, Oxford, UK) attached with the FESEM instrument. The optical properties were investigated using a UV–visible spectrophotometer (Shimadzu UV-2700, Shimadzu, Japan), in both absorbance and reflectance modes.

## Figures and Tables

**Table 1:** Estimated lattice parameter and bandgap of HEO and Pr^3+^-incorporated HEO nanoparticles prepared by gel combustion synthesis.

Sample	Lattice Parameter * (Å)	Crystallite Size ** (nm)	Micro Strain **	Bandgap *** (eV)
HEO-0	5.3668	12.3	0.00233	3.15
HEO-1	5.3682	15.3	0.00392	2.96
HEO-2	5.3699	12.8	0.00279	2.59
HEO-3	5.3749	12.4	0.00214	1.99
HEO-4	5.3776	11.8	0.00154	1.91
HEO-5	5.3804	11.5	0.00072	1.89
HEO-6	5.3857	12.1	0.00078	1.87

* Estimated from Rietveld refinement. ** Estimated from W-H plot. *** Estimated from Kubelka-Munk plot.

**Table 2:** Elemental quantification of HEO and Pr^3+^-incorporated HEO nanoparticles prepared by gel combustion synthesis using an EDS point scan.

Sample	Element (Mean Atomic Percent) *					
	Pr	Y	Zr	Ce	Sm	Gd
HEO-0	0	19.21 ± 5.12	16.96 ± 3.45	20.03 ± 2.31	24.38 ± 3.53	19.42 ± 2.64
HEO-1	0.47 ± 0.58	21.44 ± 5.00	18.34 ± 3.61	18.90 ± 2.19	22.59 ± 3.58	18.27 ± 2.61
HEO-2	2.18 ± 0.63	20.74 ± 5.08	17.59 ± 3.54	19.14 ± 2.55	22.36 ± 3.59	17.98 ± 2.42
HEO-3	4.74 ± 0.57	19.45 ± 4.00	16.91 ± 2.82	18.39 ± 1.56	22.07 ± 2.91	18.45 ± 2.19
HEO-4	7.38 ± 1.02	20.23 ± 4.48	17.32 ± 3.14	17.15 ± 1.96	20.93 ± 2.75	16.99 ± 2.17
HEO-5	11.18 ± 0.76	21.03 ± 2.22	18.44 ± 1.59	16.42 ± 1.09	17.14 ± 1.05	15.79 ± 0.95
HEO-6	16.11 ± 1.67	16.96 ± 4.55	14.83 ± 3.36	16.59 ± 1.26	19.27 ± 3.45	16.24 ± 1.74

* The mean and standard deviation values are estimated from the EDS point scan probed at 15 different points of the sample.

## Data Availability

Data are available on request from the corresponding author.

## Supplementary Materials

The following supporting information can be downloaded at: https://www.mdpi.com/article/10.3390/gels11020117/s1, Figure S1. Rietveld refinement plot of HEO and Pr^3+^ incorporated HEO nanoparticles prepared by gel combustion synthesis, calcined at 950 °C: (a) HEO-0, (b) HEO-1, (c) HEO-2, (d) HEO-3, (e) HEO-4, (f) HEO-5, and (g) HEO-6; Figure S2. W-H plot to estimate the crystallite size of HEO and Pr^3+^-incorporated HEO nanoparticles prepared by gel combustion synthesis, calcined at 950 °C: (a) HEO-0, (b) HEO-1, (c) HEO-2, (d) HEO-3, (e) HEO-4, (f) HEO-5, and (g) HEO-6.
