# Supplementary material for: Band-Gap Engineering of High-Entropy Fluorite Metal Oxide Nanoparticles Facilitated by Pr3+ Incorporation by Gel Combustion Synthesis
Source: Gels. 2025 Feb 6;11(2):117. doi: 10.3390/gels11020117 (PMC11854270; doi:10.3390/gels11020117)
Supplement: Supplementary file 1 [file gels-11-00117-s001.zip › gels-3427577-supplementary.pdf]

Supporting Information

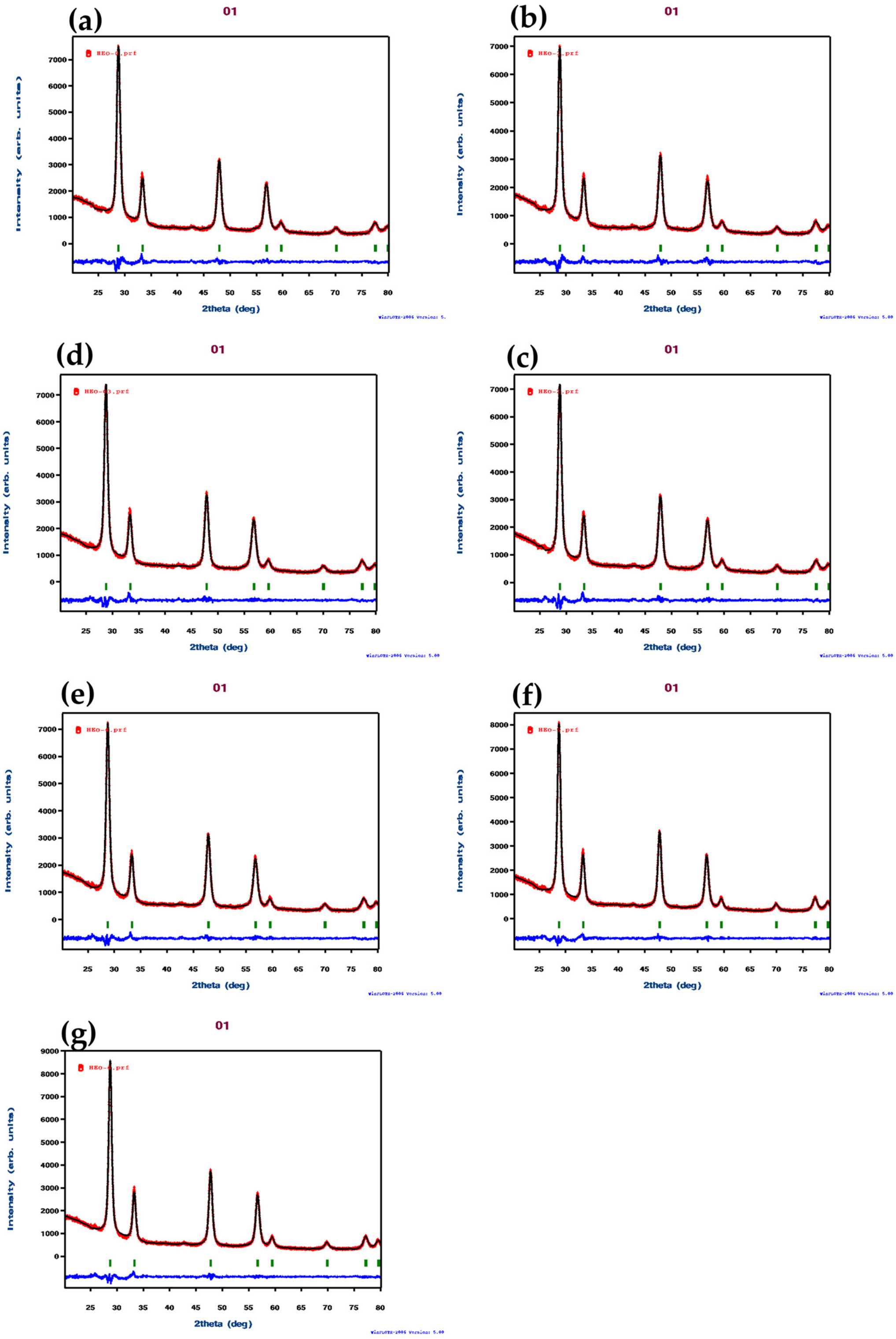

Figure S1. Rietveld refinement plot of HEO and Pr^{3+}-incorporated HEO nanoparticles prepared by gel combustion synthesis and calcined at 950 °C: (a) HEO-0, (b) HEO-1, (c) HEO-2, (d) HEO-3, (e) HEO-4, (f) HEO-5, and (g) HEO-6

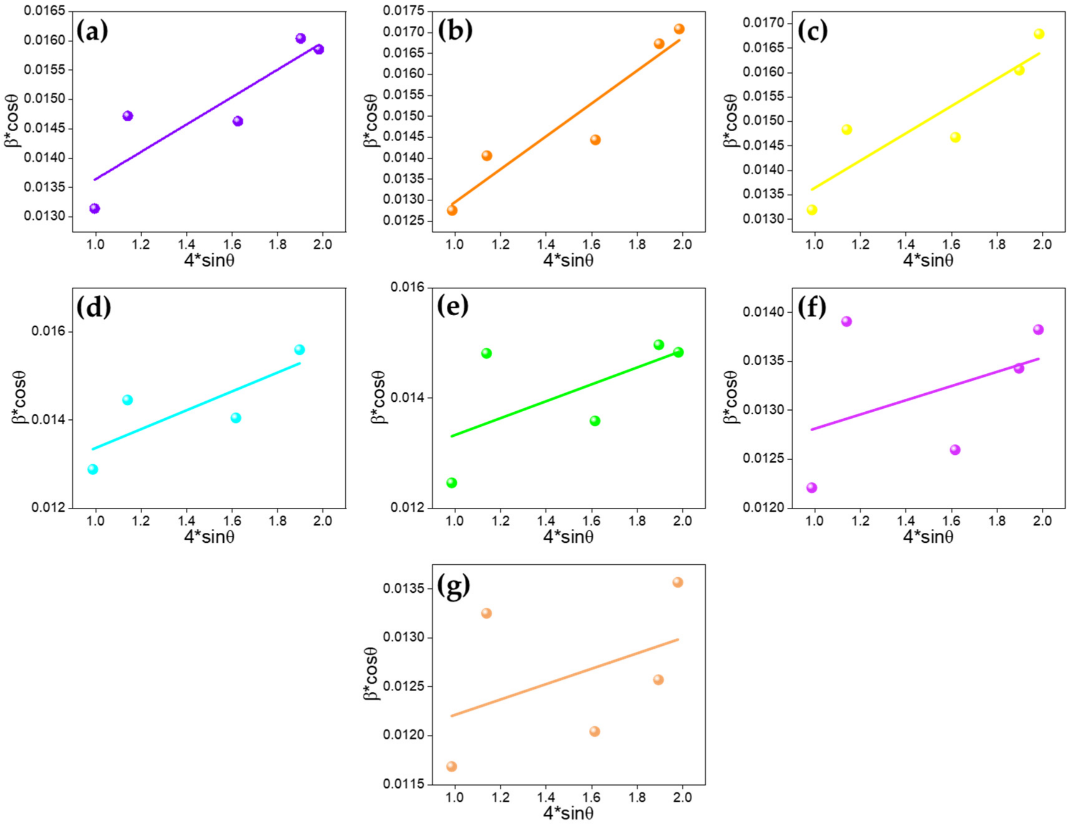

Figure S2. W-H plot to estimate the crystallite size of HEO and Pr^{3+}-incorporated HEO nanoparticles prepared by gel combustion synthesis and calcined at 950 °CM: (a) HEO-0, (b) HEO-1, (c) HEO-2, (d) HEO-3, (e) HEO-4, (f) HEO-5, and (g) HEO-6.
